# In Silico and In Vitro Study of Isoquercitrin against Kidney Cancer and Inflammation by Triggering Potential Gene Targets

**DOI:** 10.3390/cimb46040208

**Published:** 2024-04-12

**Authors:** Safia Iqbal, Md. Rezaul Karim, Shahnawaz Mohammad, Jong Chan Ahn, Anjali Kariyarath Valappil, Ramya Mathiyalagan, Deok-Chun Yang, Dae-Hyo Jung, Hyocheol Bae, Dong Uk Yang

**Affiliations:** 1 Department of Biopharmaceutical Biotechnology, College of Life Science, Kyung Hee University, Yongin-si 17104, Gyeonggi-do, Republic of Korea; safiadorin@khu.ac.kr (S.I.); rezaulshimul@khu.ac.kr (M.R.K.); anjalikv111@khu.ac.kr (A.K.V.); dcyang@khu.ac.kr (D.-C.Y.); 2 Graduate School of Biotechnology, College of Life Science, Kyung Hee University, Yongin-si 17104, Gyeonggi-do, Republic of Korea; shnwzmohd@yahoo.com (S.M.); jongchanahn7@gmail.com (J.C.A.); ramyabinfo@gmail.com (R.M.); daxiao@daum.net (D.-H.J.); 3 Department of Oriental Medicinal Biotechnology, College of Life Sciences, Kyung Hee University, Yongin 17104, Gyeonggi-do, Republic of Korea

**Keywords:** kidney cancer, kidney inflammation, isoquercitrin, MD simulation, Go analysis, KEGG pathway analysis, gene expression

## Abstract

Kidney cancer has emerged as a major medical problem in recent times. Multiple compounds are used to treat kidney cancer by triggering cancer-causing gene targets. For instance, isoquercitrin (quercetin-3-O-β-d-glucopyranoside) is frequently present in fruits, vegetables, medicinal herbs, and foods and drinks made from plants. Our previous study predicted using protein-protein interaction (PPI) and molecular docking analysis that the isoquercitrin compound can control kidney cancer and inflammation by triggering potential gene targets of IGF1R, PIK3CA, IL6, and PTGS2. So, the present study is about further in silico and in vitro validation. We performed molecular dynamic (MD) simulation, gene ontology (GO), Kyoto Encyclopedia of Genes and Genomes (KEGG) analysis, cytotoxicity assay, and RT-PCR and qRT-PCR validation. According to the MD simulation (250 ns), we found that IGF1R, PIK3CA, and PTGS2, except for IL6 gene targets, show stable binding energy with a stable complex with isoquercitrin. We also performed gene ontology (GO) and Kyoto Encyclopedia of Genes and Genomes (KEGG) analyses of the final targets to determine their regulatory functions and signaling pathways. Furthermore, we checked the cytotoxicity effect of isoquercitrin (IQ) and found that 5 μg/mL and 10 μg/mL doses showed higher cell viability in a normal kidney cell line (HEK 293) and also inversely showed an inhibition of cell growth at 35% and 45%, respectively, in the kidney cancer cell line (A498). Lastly, the RT-PCR and qRT-PCR findings showed a significant decrease in PTGS2, PIK3CA, and IGF1R gene expression, except for IL6 expression, following dose-dependent treatments with IQ. Thus, we can conclude that isoquercitrin inhibits the expression of PTGS2, PIK3CA, and IGF1R gene targets, which in turn controls kidney cancer and inflammation.

## 1. Introduction

Kidney cancer is the sixth most frequent malignancy in men and accounts for 5% of all cancer cases. In addition, it is the tenth most prevalent malignancy in women and the source of 3% of malignancies [1]. In urogenital cancer, renal cell carcinoma (RCC) is the most prevalent kind (around 90%) [2]. It is more common in men than in women and has a 30–40% fatality rate [3]. RCC makes up approximately 85% of kidney cancers, and clear cell RCC (ccRCC) accounts for about 70% of these tumors [4]. According to the Global Cancer Statistics, kidney cancer had an incidence of 431,288 cases and a mortality rate of 179,368 cases in 2020 [3]. According to the American Cancer Society’s most current projections, there will be around 81,610 new cases of kidney cancer identified in the US in 2024 (52,380 in men and 29,230 in women) [5]. So, an increasing number of cases indicate that kidney cancer is a topic of concern in the modern world. Our earlier in silico investigation revealed the role of an IQ compound in the regulation of kidney cancer and inflammation [6].

One of the main glycosidic forms of the naturally occurring flavonoid quercetin (3,5,7,3′,4′-pentahydroxyflavone) is isoquercitrin (quercetin-3-O-b-D-glucopyranoside), along with rutin (quercetin-3-O-rutinoside) [7]. During in vitro analysis, IQ has been shown repeatedly to exhibit antioxidant activity by reducing the mortality of cells caused by oxidative stress. In isolated human lymphocytes, for example, isoquercitrin reduced H_2_O_2_-induced DNA damage [8]. Jung et al. (2010) reported that H_2_O_2_ treatment also decreased lipid peroxidation, intracellular ROS levels, and glutathione depletion in rat retinal ganglion RGC-5 cells [9]. It should be noted that isoquercitrin can, like other flavonoids, show pro-oxidant behavior due to auto-oxidation, which creates reactive flavonoid phenoxyl radicals or oxidation of flavonoids by ROS or peroxidases [10]. Therefore, it is widely accepted that the antioxidant properties of flavonoids may be beneficial for several oxidative stress-related disorders, including cancer and inflammation [11]. For instance, IQ can be regulated by suppressing colon cancer cell growth by triggering the Wnt/β-catenin signaling pathway [12]. Furthermore, IQ regulates opioid receptors and the mitogen-activated protein kinase signaling pathway to prevent pancreatic cancer from progressing both in vivo and in vitro [7]. Despite isoquercitrin’s many amazing health benefits, its exact mechanism of action for kidney cancer and inflammation is yet unknown to us.

**Figure 1:**
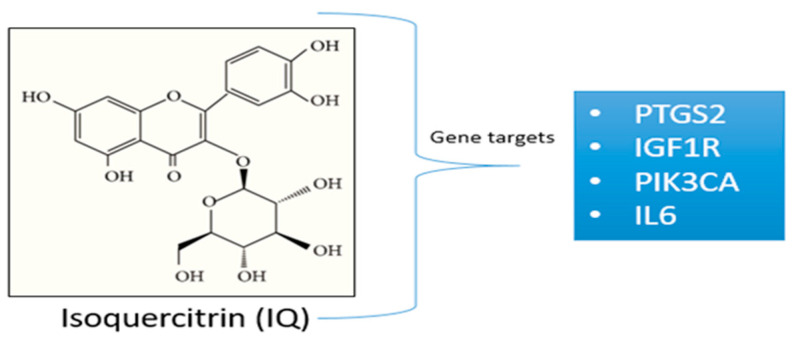
Chemical structure of isoquercitrin and its gene targets.

Moreover, the majority of human diseases, including cancer, have pathological processes that are primarily caused by altered gene expression [13]. Our previous in silico protein–protein interaction (PPI) and molecular docking studies revealed that the isoquercitrin compound could trigger potential cancer-causing gene targets, such as IGF1R, PIK3CA, IL6, and PTGS2, which can regulate kidney cancer and inflammation (Figure 1). We considered different databases and in silico platforms to perform our previous studies. In the present studies, we performed further in silico and in vitro validation of isoquercitrin efficacy against kidney cancer and inflammation by suppressing the expression of the mentioned gene targets.

## 2. Materials and Methods

### 2.1. Selection of Gene Targets and Molecular Docking Study

In our prior study, we simulated molecular docking using the AutoDock Vina Wizard using PyRx as the default option [6]. After the molecular docking assay, we predicted four gene targets (IGF1R, PIK3CA, PTGS2, and IL6) that can be triggered by the isoquercitrin compound to control kidney cancer and inflammation.

### 2.2. Molecular Dynamic (MD) Simulation

The thermodynamic integrity of receptor–ligand interactions was assessed by dynamically simulating the interaction using the “Desmond v6.3 Program” in Schrödinger 2023-3 on the Linux platform [14]. To find how well the isoquercitrin molecule bonded to the target protein and its active site cavity, MD simulation (250 ns) was run for the ranked gene protein targets (IGF1R, PIK3CA, PTGS2, and IL6). A predefined TIP3P water model was used to solve the system by preserving a specific volume using an orthorhombic periodic boundary box that was spaced 10 Å on both sides. Both sodium and chlorine ions were used to neutralize the system and lower the salt level to 0.15 M molar. There was a neutralization of the 9 Cl^−^ and 11 Na^+^ ions. Using the force field parameters of OPLS_2005 and the default protocol applied to the Desmond module, the solvated system containing the protein and binding ligand was decreased and relaxed. The NPT ensemble was kept at 310 K using the Nose–Hoover (NH) thermal coupling with isotropic scaling technique and a single standard pressure of 1.01325 bar. Next, we recorded at 1.2 energy using 250 PS recording frequencies. According to Baral et al. (2021) [15], the highest temperatures recorded for non-Newtonian and Newtonian blood flies are 310.007 K and 310.0045 K, respectively. According to Mahdavi et al. (2020) [16], 310 K is the temperature at which the system is most impacted. Using Maestro v-12.5, all MD simulation snapshots were produced. Based on the 250 ns trajectory performance, the stability of the complex structure was evaluated using the Desmond module v6.3 simulation interaction diagram (SID), root-mean-square fluctuation (RMSF), root-mean-square deviation (RMSD), solvent-accessible surface area (SASA), ROG (radius of gyration), hydrogen bond interaction, and P-L contact (protein–ligand contacts).

### 2.3. Investigation of Fee-Binding Energy by Prime MM-GBSA

Molecular mechanics, the Generalized Born model, and solvent accessibility (MM-GBSA) study were used to investigate the protein and ligand complexes’ free binding energies. Using the docked posture obtained from the Glide method, the Prime MM-GBSA algorithm was utilized to determine the free energy of binding (ΔG bind) for the ligand molecules. The Prime/MM–GBSA method based on the docking complex was used to calculate the binding-free energy (ΔG bind) of each ligand, using the following equation [17]:

ΔG (bind) = ΔG (solv) + ΔG (SA) + ΔE (MM)

ΔG (solv) represents the total solvation energy, as well as the variation in GBSA solvation and the complex between the ligand and protein. The total surface area energies and the difference in the surface area energies of the protein and ligand complex are denoted by ΔG (SA). The total energy and the difference between the minimized energies in the protein and ligand complex is known as ΔE (MM).

The use of “Molecular Biomechanics Generalized Born Surface Area” (MMGBSA) techniques has grown in popularity as a means of assessing ligands’ capacity to bind their free energy to macromolecules [18]. Using the Prime MM-GBSA with default values, the Maestro program provided in Schrödinger (Release 2020-3) was used to calculate the bound free energy for the compounds.

### 2.4. Kyoto Encyclopedia of Genes and Genomes (KEGG) and Gene Ontology (GO) Databases

GO analysis was performed to define the gene function analysis, including biological function (BF), molecular functions (MF), and cellular components (CC). Moreover, KEGG pathways analysis can be used to predict the signaling pathways that can be influenced by the gene targets [19]. GO and KEGG pathways analysis of selective targets were analyzed by the Protein Analysis Through Evolutionary Relationships (PANTHER) online-based software v18.0 (http://pantherdb.org/, accessed on 17 February 2024) [20].

### 2.5. Cell Culture

As a normal kidney cell line, human embryonic kidney cells (HEK 293) were cultured in a medium that included 86% Dulbecco’s Modified Eagle Medium (DMEM/F12) (SolBio, Suwon-si, Republic of Korea), 10% Fetal Bovine Serum (FBS), and 1% penicillin–streptomycin. A medium comprising 89% Minimum Essential Medium (MEM) (WELGENE, Fresh Media, Gyeongsan-si, Republic of Korea), 10% Fetal Bovine Serum (FBS), and 1% penicillin–streptomycin was used to cultivate A498 kidney cancer cells. Both cell lines were cultured in a carbon dioxide (CO_2_) chamber at 37 °C for a whole day after those cells were prepared for treatment.

### 2.6. Cell Viability, Proliferation, and Cytotoxicity Measured by MTT Assay

We used an MTT solution to check the cytotoxicity of isoquercitrin (purity ≥ 98%, Chem Faces, Cat No. CFN98753, Wuhan, China) in both HEK 293 and A498 cell lines. As a standard, we used cisplatin (10 µg/mL) in the A498 cell line only to check the efficacy and compare it with the isoquercitrin treatment. We utilized 96-well plates for the cell treatment, plating 1 × 10^4^ cells/well of HEK 293 and A498. Subsequently, isoquercitrin was applied to both cell lines at several doses (0.75, 1.25, 2.5, 5, 10, and 25 µg/mL). Following that, it was incubated for 24 h. After 24 h, the cells were treated with 20 µL of 3-(4,5-dimethyl-2-thiazolyl)-2,5-diphenyl tetrazolium bromide solution (MTT; 5 mg/mL, in PBS; Life Technologies, Eugene, OR, USA) and incubated for 3–4 h at 37 °C. However, the formation of purple-colored formazan occurred in live cells due to the addition of the MTT reagent. So, 100 µL of DMSO was added to each well to dissolve the insoluble formazan agents. Finally, the data were obtained at a wavelength of 570 nm using an ELISA reader (BioTek Instruments, Inc., Winooski, VT, USA).

### 2.7. In Vitro Validation by Reverse Transcription Polymerase Chain Reaction (RT-PCR)

After the treatment with isoquercitrin, the total RNA from A498 cells was extracted using a lysis reagent (QIAzol, from QIAGEN, Hermantown, MD, USA), and then the RT-PCR process was performed by using 1 µg of RNA in 20 µL of RT-PCR premix (amfiRivert, GenDepot, Barker, TX, USA), primer, purified nuclease-free water, and buffer mixture. For RT-PCR we used the following primers: PIK3CA, forward: 5′-AAAGATAACTGAGAAAATGAAAGCTC-3′, and reverse: 5′-GAAGAAAGCTGACCATGCTGCTATG-3′; IGF1R, forward: 5′-GCTTCTGTGAACCCCGAGTATTT-3′, and reverse: 5′-TGGTGATCTTCTCTCGAGCTACCT-3′; PTGS2, forward: 5′-CCCTTGGGTGTCAAAGGTA-3′ and reverse: 5′-TGGTGATCTTCTCTCGAGCTACCT-3′; and IL6, forward: 5′-TGGTGATCTTCTCTCGAGCTACCT-3′, and reverse: 5′-TCTGAAGAGGTGAGTGGCTGTC-3′, GAPDH, 5′-ATGGTGAAGGTCGGTGTGAAC-3′, and reverse: 5′-TTGATGTTAGTGGGGTCTCGC-3′. The RT-PCR was carried out at 95 °C (30 s for denature), 60 °C (30 s for primer annealing), and 72 °C (50 s for elongation), and all processes were repeated for 38 cycles. Finally, we visualized the RT-PCR results on 1% agarose gel electrophoresis, and the picture was captured under a UV chamber.

### 2.8. qRT-PCR Assay Validation

We used SYBR TOPreal qPCR2X Premix (Enzynomics, Daejeon, Republic of Korea) to conduct qRT-PCR. The qRT-PCR reaction was performed in three replicates, and the reaction volume was 20 µL (10 µL TOPreal qPCR2X master Premix, 1 µL of each primer mentioned in the RT-PCR section, 1 µL of cDNA template, and the rest of nuclease-free water. To perform qRT-PCR the following condition was applied: 95 °C for 5 min, then 38 cycles of 95 °C for 10 s and 55–60 °C for 45 s, followed by 15 s at 72 °C. Using the comparative 2^−∆∆Ct^ technique methods to calculate the expression and to normalize it, we used the expression of the GAPDH gene. All qRT-PCR analysis was performed in the aCFX Connect Real-Time PCR (Bio-Rad, Hercules, CA, USA).

## 3. Results

### 3.1. Gene Targets from the Previous Study

In our previous study, we predicted that four (4) different gene targets (IGF1R, PIK3CA, PTGS2, and IL6) could be triggered by the isoquercitrin compound (Table 1). We predicted this via in silico-based network pharmacology analyses such as datasets prepared from different databases (both diseases gene targets and also compound-based gene targets), Venn diagrams, protein-protein interactions (PPI), and molecular docking [6]. In the present study, we performed further in silico and in vitro validation to check the efficacy of isoquercitrin against kidney cancer and inflammation.

### 3.2. In Silico Molecular Dynamic (MD) Simulation

**Figure 2:**
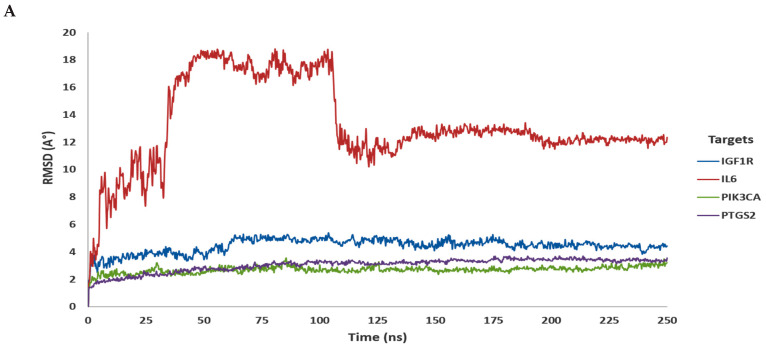

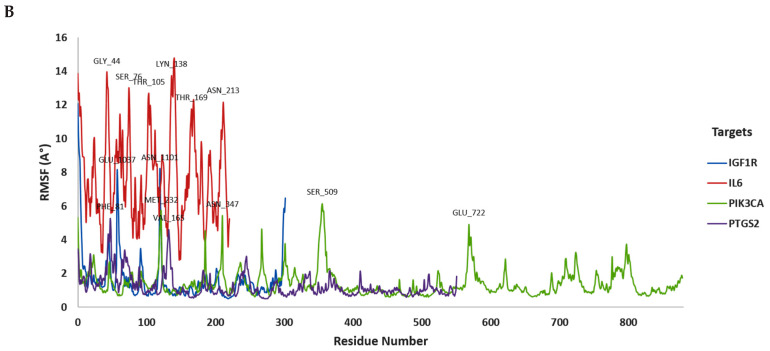
Molecular dynamic simulation (Part-1). (**A**) RMSD and (**B**) RMSF. RMSD—root-mean-square deviation; RMSF—root-mean-square fluctuation. (Desmond v6.3 Program in Schrödinger 2023-3 under the Linux platform was used for this simulation).

To check the protein structure stability during the 250 ns simulation, we calculated the root-mean-square deviation (RMSDs) of Cα atoms of complexes between isoquercitrin and four different genes (IGF1R, PIK3CA, PTGS2, and IL6) targets. For the isoquercitrin and IGF1R complex, after 75 ns, the complex was very stable; the lowest and highest RMSD values were found at 3.839 Å and 5.402 Å, respectively. The fluctuation rate was relatively low on average (0.755 Å). The results showed that the RMSD for 250 ns was 4.43 Å on average, 5.402 Å at maximum, and 1.922 Å at lowest (Figure 2A). For isoquercitrin and PIK3CA, the complex was stable after 90 ns; the lowest and highest RMSD values were found to be 2.36 Å and 3.484 Å, respectively. The fluctuation rate was relatively low on average (0.715). For 250 ns, the maximum, lowest, and average RMSDs were found to be 2.70 Å, 3.556 Å, and 1.349 Å, respectively (Figure 2A). Other complexes were isoquercitrin and PTGS2; the complexes were stable after 80 ns, and the lowest and highest RMSD values were found to be 2.885Å and 3.693 Å, respectively. The average fluctuation rate was very low (0.379 Å). During 250 ns, the average, maximum, and lowest RMSD were found to be 3.039 Å, 3.693 Å, and 1.24 Å, respectively (Figure 2A). The fourth complex included isoquercitrin and IL6, which fluctuated at high rates (5.514 Å) during 250 ns of simulation. The average, highest, and lowest RMSD values were 13.267 Å, 18.781 Å, and 1.721 Å, respectively (Figure 2A). Thus, every complex was stable except the IL6 complex (Supplementary S1–S4).

RMSF measures protein structural variations in amino acid (AA) residues. The degree of stability and variation of amino acid residues in a compound–protein complex system is predicted by their high RMSF value. For the Isoquercitrin and IGF1R complex, during the simulation period, the average, lowest, and highest values of RMSF were 1.596 Å, 0.491 Å, and 10.049 Å, respectively (Figure 2B). In the isoquercitrin and PIK3CA complex, the average, lowest, and highest RMSF were 1.403 Å, 0.592 Å, and 6.127 Å, respectively (Figure 2B). Furthermore, the isoquercitrin and PTGS2 complex showed that the average, lowest, and highest RMSF were 1.229 Å, 0.49 Å, and 5.253 Å (Figure 2B). But the isoquercitrin and IL6 complex showed many fluctuations in many amino acid residues (GLY_44, SER_76, THR_105, LYN_138, THR_169, and ASN_213), and showed the average, lowest, and highest RMSF values were 7.782 Å, 2.842 Å, and 14.754 Å (Figure 2B). So, here, we also found that all isoquercitrin with gene target complexes were stable except for IL6 (Supplementary S1–S4).

**Figure 3:**
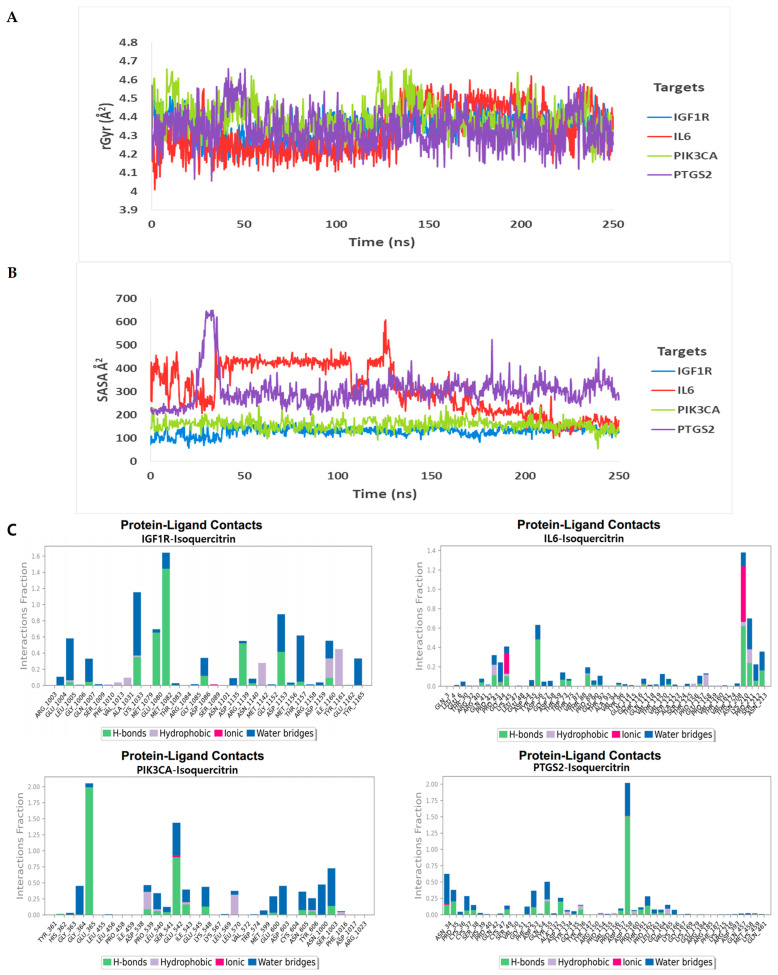
Molecular dynamic simulation (Part 2): (**A**) radius of gyration (rGyr); (**B**) solvent–accessible surface area (SASA); and (**C**) protein–ligand contact from the MD simulation trajectory. (Desmond v6.3 Program in Schrödinger 2023-3 under the Linux platform was used for this simulation).

In addition, rGyr, SASA [16], and protein-ligand contact from the MD simulation trajectory were analyzed for stable protein–ligand complexes (Figure 3A–C). The rGyr and SASA exhibited good stability, and their results were the opposite, except for the isoquercitrin–IL6 complex (Figure 3A,B). We observed that the values of rGyr and SASA for the isoquercitrin–IL6 complex fluctuated from the beginning of the MD simulation (Figure 3A,B). The average rGyr values were observed at 4.350 Å^2^ (isoquercitrin–IGF1R), 4.398 Å^2^ (isoquercitrin–PIK3CA), 4.335 Å^2^ (isoquercitrin–PTGS2), and 4.333 Å^2^ (isoquercitrin–IL6). Furthermore, the average SASA values were observed at 130.083 Å^2^ (isoquercitrin–IGF1R), 157.610 Å^2^ (isoquercitrin–PIK3CA), 304.199 Å^2^ (isoquercitrin–PTGS2), and 309.659 Å^2^ (isoquercitrin–IL6). So, the rGyr and SASA results confirmed the stability of all three complexes except for the isoquercitrin–IL6 complex. Moreover, the complexes between all proteins with the ligand were examined (Figure 3C). There, we observed hydrogen bonds, non-covalent bonds, ionic bonds, and water bridge bonds. Except for the isoquercitrin and IL6 complex, all complexes showed a higher fraction of hydrogen bonds in different amino acid residues. So, all MD simulation results indicate stable complexes between isoquercitrin and three gene targets (IGF1R, PIK3CA, and PTGS2) (Supplementary S1–S4).

### 3.3. Molecular Mechanics-Generalized Born Surface Area (MM-GBSA) Analysis

The binding energies were estimated using the VSGB solvation model and the MM-GBSA protocol, utilizing the Schrödinger software’s (2023-3) Glide module. To eliminate misleading positive predictions, we performed MM-GBSA rescoring. We discovered each complex’s free-binding energy. Details on the complexes and individual contributions to the total energy values are given in Table 2. Additionally, we found the total free-binding energy for each complex, such as isoquercitrin-IGF1R (−40.41 kcal mol^−1^), isoquercitrin-PIK3CA (−54.81 kcal mol^−1^), isoquercitrin-PTGS2 (−42.60 kcal mol^−1^), and isoquercitrin-IL6 (−21.82 kcal mol^−1^). Like total free energy, most of the interaction bond energy was lower in the isoquercitrin–IL6 complex (Table 2). So, the other complexes are shown to be more stable than the isoquercitrin–IL6 complex.

### 3.4. Gene Function and Pathways Regulated by Selected Gene Targets

**Figure 4:**
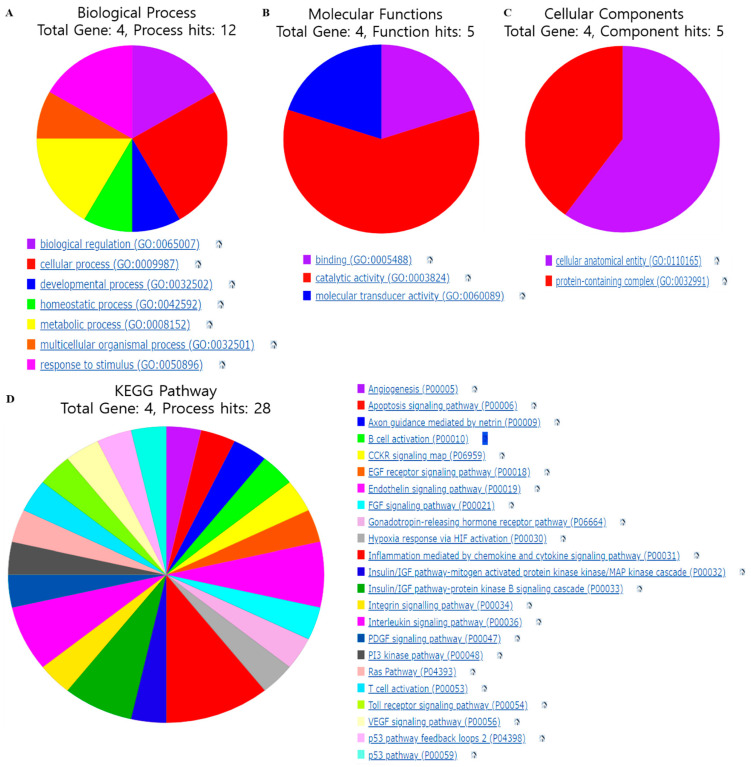
Gene ontology (GO) and KEGG pathway analysis of the target genes. (**A**) Biological process. (**B**) Molecular functions. (**C**) Cellular components. (**D**) KEGG pathway analysis.

To further investigate the potential mechanism of the genes for kidney cancer and inflammation, GO, KEGG, and enrichment analyses were conducted based on four targets: IGF1R, PIK3CA, PTGS2, and IL6. We observed that the four (four) gene targets were involved in twelve biological processes (BPs) (metabolic processes, biological regulation, cellular processes, and response to stimulus were the main biological processes), five molecular function (MF) terms (catalytic activity, molecular transducer activity, and binding function were the main functions), and also five cellular component (CC) terms (mainly involved in protein-containing complex and cellular anatomical entity) for causing kidney cancer and kidney inflammation. So, we found the top seven entries for BPs, three entries for MFs, and two entries for cellular components in terms of GO analysis to control kidney cancer and inflammation (Figure 4A–C). Additionally, via KEGG pathway analysis, we observed that the targeted genes were involved in 28 regulating pathways, primarily the interleukin signaling pathway, the insulin/IGF pathway–protein kinase B signaling cascade, the cytokine signaling pathway, the endothelin signaling pathway, and inflammation mediated by chemokines (Figure 4D).

### 3.5. Cytotoxicity Effects of Isoquercitrin

**Figure 5:**
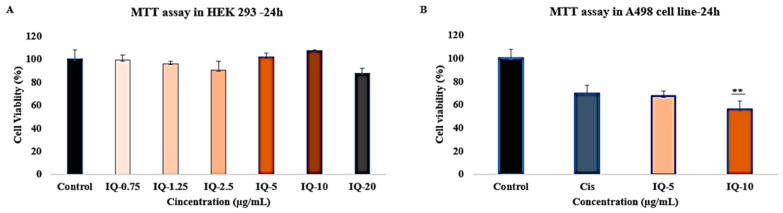
MTT assay to determine cell viability percentage. (**A**) HEK 293 normal kidney cell treatment with different doses of isoquercitrin (IQ); (**B**) A493 kidney cancer cell line treated with cisplatin and isoquercitrin (IQ). ** *p* < 0.001 indicates significant differences from control groups.

IQ with different gene targets can control kidney cancer and inflammation. To validate the in silico prediction, we performed in vitro analysis. For effective dose selection, firstly, we performed an MTT assay in the HEK 293 normal kidney cell line and found that cell viability showed a lower toxicity percentage in comparison to the control at doses of 5 and 10 μg/mL (Figure 5A). Furthermore, we tested the A498 kidney cancer cell line with two different doses of isoquercitrin and found that isoquercitrin can reduce the kidney cancer cell proliferation by around 35% at a concentration of 5 μg/mL and 45% at a concentration of 10 μg/mL. On the other hand, cisplatin was employed as a positive control and decreased proliferation by about 31%, which is lower than IQ (Figure 5B and Figure S1).

### 3.6. Isoquercitrin Controls the Gene Expression of the Selective Gene Targets

**Figure 6:**
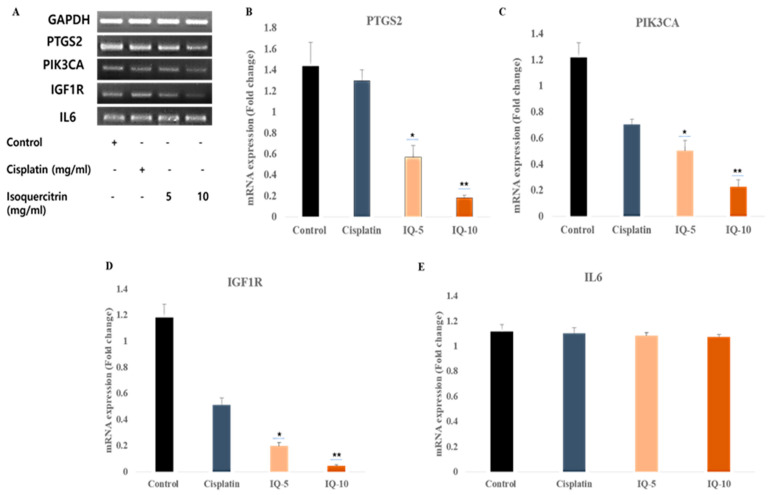
Impacts of isoquercitrin (IQ) and cisplatin on the cDNA expression levels of genes linked to kidney cancer and inflammation in A498 cells. (**A**) RT-PCR and (**B**–**E**) qRT-PCR expression profile for targeted genes (control: without treatment, cisplatin: 10 μg/µL; and doses of IQ of 5 μg/µL and 10 μg/µL were applied for 24 h. Following the extraction of total RNA, we prepared cDNA then performed RT-PCR and qRT-PCR). For qRT–PCR, all results display the mean ± SE of duplicate samples from 3 independent experiments (* *p* < 0.05 and ** *p* < 0.01 using Student’s *t*-test compared to the non-treated control).

To observe and confirm the putative inhibitory action of IQ, we first used RT-PCR to measure the level of RNA expression in the A498 kidney cancer cell line. We found that both 5 µg/µL and 10 µg/µL of IQ could significantly suppress PTGS2, PIK3CA, and IGF1R gene targets except IL6. This finding validated the simulation results as well (Figure 6A). Here, we used cisplatin as a control drug and compared it with the isoquercitrin treatment. Among the two doses of isoquercitrin, 10 µg/µL showed the maximum inhibition.

Furthermore, we also performed qRT-PCR for the dose-dependent evaluation of different target genes expression (Figure 6B–E). Our study revealed that the mRNA expression of PTGS2, PIK3CA, and IGF1R genes were downregulated in a dose-dependent manner. We observed that the PTGS2 gene expression profile, during negative control (without any treatment), showed an expression of PTGS2 (1.43-fold), whereas cisplatin treatment showed downregulation (1.29-fold), and isoquercitrin (5 µg/mL and 10 µg/µL) treatment showed significantly downregulated (0.57- and 0.18-fold, respectively). Consequently, we observed that PIK3CA and IGF1R gene expressions showed almost the same expression status. For PIK3CA, after treatment with IQ, the expression was significantly downregulated as compared to without treatment and the control drug (1.22-fold: without treatment, 0.71-fold: cisplatin, 0.5-fold: IQ 5 µg/mL, and 0.23-fold: IQ 10 µg/mL). Similarly, IGF1R expression was significantly suppressed compared to without treatment and control drug (1.12-fold: without treatment, 0.51-fold: cisplatin, 0.20-fold: IQ 5 µg/mL, and 0.0-fold: IQ 10 µg/mL). However, all treatments did not show inhibition of IL6 mRNA expression. So, all results validated the finding that isoquercitrin treatment can suppress PTGS2, PIK3CA, and IGF1R expression and thus control kidney cancer and inflammation.

## 4. Discussion

The prevalence of malignant kidney tumors is rising, making up 2% of all cancer cases worldwide [21]. According to the Global Cancer Statistics in 2020, kidney cancer had an incidence of 431,288 cases and a fatality rate of 179,368 cases [3]. Recently, the control of kidney cancer and inflammation has been a major concern. The majority of the genes involved in hereditary renal cell carcinoma (RCC) are HRPT2, FH, BHD, MET, VHL, and FH. The VHL gene is the most often occurring site of mutation that results in hereditary clear cell RCC [22]. In our previous study, we also found that PTGS2, PIK3CA, IGF1R, and IL6 commonly caused kidney cancer and inflammation, and by suppressing these genes, we can reduce the severity of kidney cancer and inflammation. Each of these genes has a chance of developing a certain cancer. For example, the PTGS2 gene target is highly expressed in colorectal cancer [23], and is also highly expressed in colorectal cancer [24,25]. Furthermore, PIK3CA is a marker gene that is highly expressed in several cancer types, including breast, colon, ovarian, gastric, lung, brain, skin, and so on [9]. Similarly, IGF1R is our other chosen target; it is also strongly expressed in many different cancer types, including ovarian, breast, uterine, stomach, skin, lung, and adrenocortical [26,27]. Furthermore, numerous studies have demonstrated the unusually high activation of the IL-6/JAK2/STAT3 signaling pathway in a range of malignancies, including gastric, breast, liver, colorectal (CRC), colon, ovarian (OC), lung, and pancreatic cancers [28].

We also predicted that isoquercitrin (the most ubiquitous flavonoid) could trigger these four targets. This compound also demonstrates many beneficial effects, both in vitro and in vivo. These effects include chemoprotective properties against oxidative stress, cancer, cardiovascular disease, diabetes, and allergic reactions [29]. But our prediction was fully in silico-based. So, for further validation purposes, we performed additional in silico- and in vitro-based research.

Therefore, we performed an MD simulation (250 ns) to confirm and identify the stable complex between IQ and gene targets (PTGS2, PIK3CA, IGF1R, and IL6). Furthermore, the MD simulation confirmed a protein’s integrity during a ligand–protein interaction [30]. The complexes between compound IQ and PTGS2, PIK3CA, and IGF1R displayed strongly stable RMSD and RMSF values, as per MD modeling. The center of mass calculated for ROG, which is derived from the protein C and N terminal, looks at the protein’s structural integrity and gives us a better understanding of the properties of protein folding [30]. A greater ROG score denotes material detachment from the protein, but a lower ROG value suggests excellent compactness [31]. The results indicate that compound IQ has a greater ROG value with IL6 complex than other complexes. Our MD simulation study revealed that the complex between IQ with PTGS2, PIK3CA, and IGF1R is more stable than IL6. MM-GBSA calculation also confirmed the same results. Prime-MMGBSA analysis also indicates the strong complex between IQ with PTGS2, PIK3CA, and IGF1R.

Furthermore, we performed in vitro validation of IQ efficacy using RT-PCR and qRT-PCR assays and confirmed that IQ can trigger PTGS2, PIK3CA, and IGF1R gene targets to control kidney cancer and inflammation. The expression level of these genes significantly decreases during treatment with IQ.

## 5. Conclusions

In summary, IQ can trigger the gene targets of PTGS2, IGF1R, and PIK3CA, which in turn regulate kidney cancer and inflammation. To validate the screening targets produced from these in silico and in vitro analyses, we will carry out more animal and clinical studies in the future to provide further scientific data in support of the therapeutic use of IQ in the treatment of kidney cancer and inflammation.

## Figures and Tables

**Table 1:** Molecular docking-based prediction of isoquercitrin and selective gene target complexes against kidney cancer and inflammation [6].

Compound	Gene Target	Docking Score	Hydrogen Bond	Other Bonds	Docking Structure
**Isoquercitrin**	**PTGS2**	−9.5	TYR 136, ASN 34	PRO 153, CYS 36, ASP 133	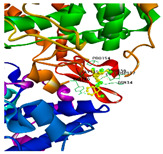
	**PIK3CA**	−8.4	GLN 682, LYN 678, SER 464, ASN 428	ASP 133	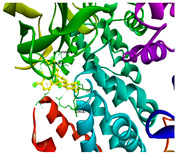
	**IGF1R**	−7.9	MET 1082, MET 1156, THR 1083	GLY 1085, ARG 1084, LEU 1005, SER 1089	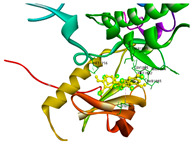
	**IL6**	−7.9	ALA 177, GLN 144, GLY 43, THR 44	VAL 94, GLU 157, PRO 176	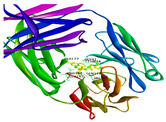

**Table 2:** The free energy (ΔG) parameters and the binding scores of various isoquercitrin complexes were calculated by the MM-GBSA algorithm using the docked pose retrieved from the Glide algorithm.

Complex	Isoquercitrin–IGF1R	Isoquercitrin–PIK3CA	Isoquercitrin–PTGS2	Isoquercitrin–IL6
Total ΔG Bind (NS) Kcal/mol	−40.41	−54.81	−42.60	−21.82
ΔG Bind(NS) Coulomb Kcal/mol	−27.18	−44.49	−58.85	−20.61
ΔG Bind Covalent Kcal/mol	12.21	5.09	9.90	5.13
ΔG Bind(NS) Hbond Kcal/mol	−2.55	−6.21	−4.74	−1.99
ΔG Bind(NS) Lipo Kcal/mol	−12.87	−12.50	−7.34	−4.84
ΔG Bind(NS) vdW Kcal/mol	−40.03	−46.01	−32.44	−25.80
ΔG Bind(NS) Solv GB Kcal/mol	30.01	49.82	50.94	28.18
Ligand stain energy Kcal/mol	20.952	8.794	17.008	12.189

## Data Availability

All data generated or analyzed during this study are included in this published article (and its Supplementary Information Files).

## Supplementary Materials

The following supporting information can be downloaded at https://www.mdpi.com/article/10.3390/cimb46040208/s1. Supplementary S1. Simulation results of PTGS2 gene target and IQ complex; Supplementary S2. Simulation results of PIK3CA gene target and IQ complex; Supplementary S3. Simulation results of IGF1R gene target and IQ complex; Supplementary S4. Simulation results of IL6 gene target and IQ complex. Figure S1. Effect of different compounds on A498 kidney cancer cell line. (A) Before treatment (cultured for 24 h); (B) After treatment with cisplatin (10 μg/mL-24 h); (C,D) After treatment with IQ (two doses 5 μg/mL, and 10 μg/mL respectively-24 h).
