# Supplementary material for: In Silico and In Vitro Study of Isoquercitrin against Kidney Cancer and Inflammation by Triggering Potential Gene Targets
Source: Curr Issues Mol Biol. 2024 Apr 12;46(4):3328–41. doi: 10.3390/cimb46040208 (PMC11049307; doi:10.3390/cimb46040208)
Supplement: Supplementary file 1 [file cimb-46-00208-s001.zip › Figure S1.pdf]

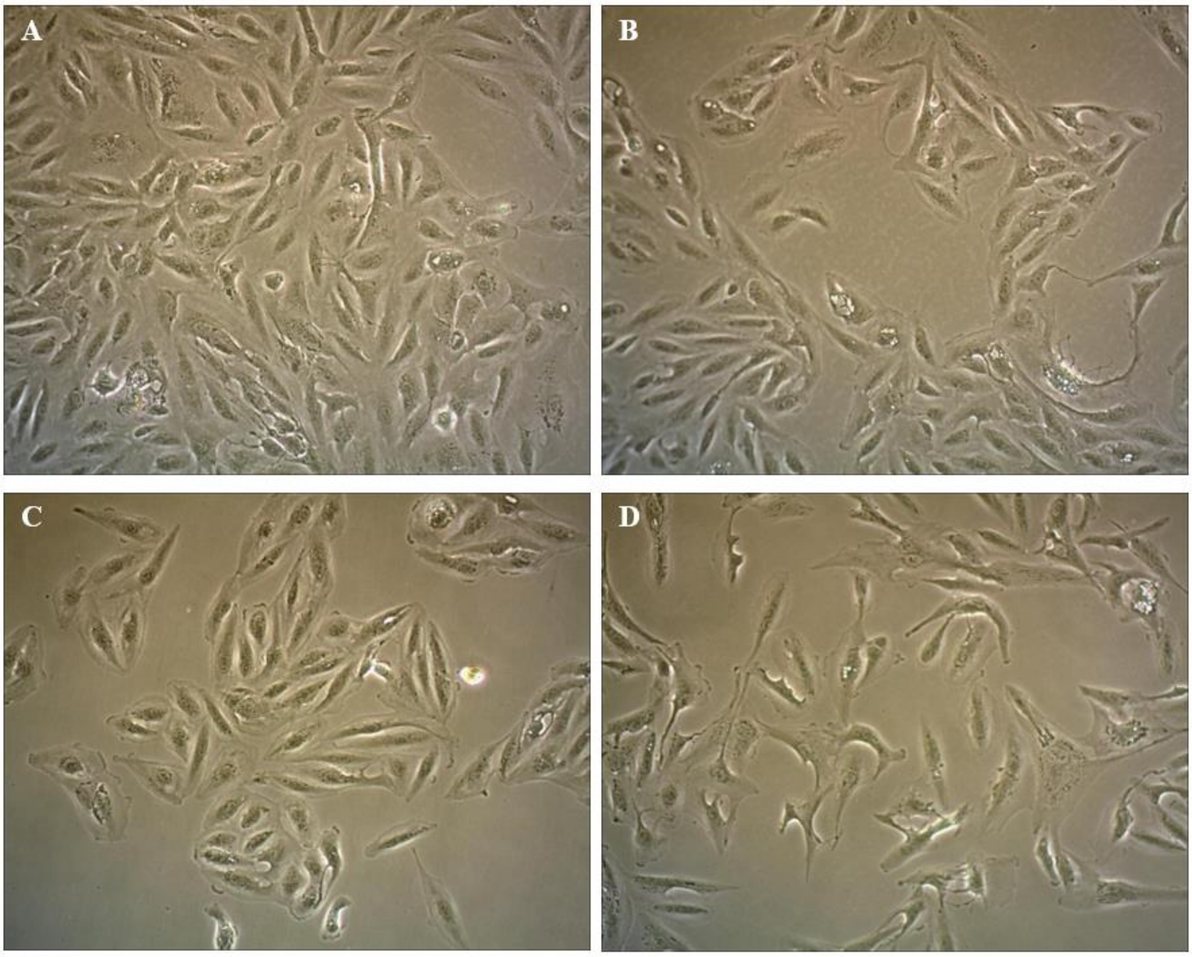

Figure S1: Effect of different compounds on A498 kidney cancer cell line. A. Before treatment (cultured for 24 hours), B. After treatment with cisplatin (10 μg/ml- 24 hours), C-D. After treatment with IQ (two doses 5 μg/ml, and 10 μg/ml respectively – 24 hours).
